# A dual-functional Ta/TaO_*x*_/Ru device with both nonlinear selector and resistive switching behaviors

**DOI:** 10.1039/d1ra02350k

**Published:** 2021-05-20

**Authors:** Rui Wang, Tuo Shi, Xumeng Zhang, Zuheng Wu, Qi Liu

**Affiliations:** The Key Laboratory of Microelectronics Device and Integrated Technology, Institute of Microelectronics, Chinese Academy of Sciences Beijing 100029 China shituo@ime.ac.cn; University of Chinese Academy of Sciences Beijing 100049 China; The Frontier Institute of Chip and System, Fudan University Shanghai 200433 China; Institute of Artificial Intelligence, Zhejiang Laboratory Hangzhou 311122 China

## Abstract

In this work, we demonstrate that a Ta/TaO_*x*_/Ru device can act as both a highly uniform and nonlinear selection device and a stable resistive switching device, respectively, by controlling the voltage applied to the Ta electrode. As a selection device, it shows high selectivity (10^3^), high current density (25 kA cm^−2^), very low variation, and good endurance. The non-linear performance of the device may be attributed to a trapezoidal band structure modulated by the concentration gradient of oxygen vacancies. Furthermore, with a large voltage bias on the Ta electrode, a repeatable and stable resistive switching behavior was observed, which could be attributed to the formation of conductive filaments probably composed of Ta metal and oxygen vacancies. This research deepens the understanding of the mechanism of Ta/TaO_*x*_ devices, and provides a potential solution for large-scale memristor arrays.

## Abstract 2

We presented a dual-functional Ta/TaO_*x*_/Ru device with both highly uniform nonlinear selector and stable resistive switching behaviors.

## Graphical abstract

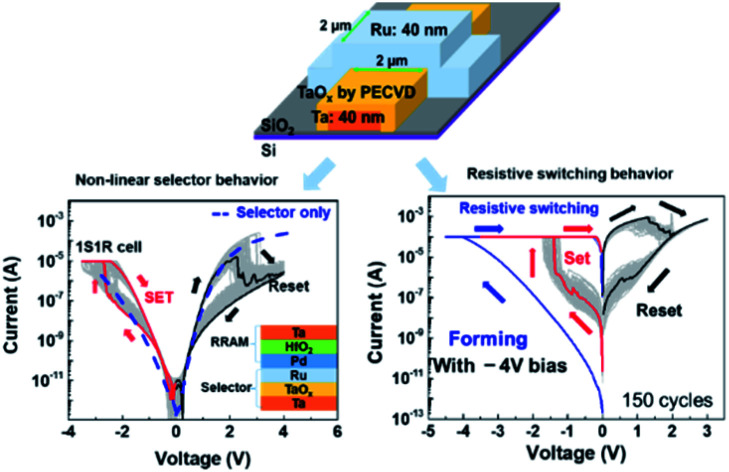

## Introduction

The memristor has been recognized to be a promising candidate for non-volatile memory due to a simple metal/insulator/metal (MIM) structure, fast speed, low power consumption and high integration density.^1–3^ The crossbar array architecture is a very effective and simple means to achieve high-density integration with a small memory-cell size of ≦4*F*^2^.^4,5^ Due to the direct accomplishment of dot products through Ohm's and Kirchhoff's laws, the memristor crossbar arrays are very suitable for some specific applications, for example, neuromorphic computing systems.^6–11^ However, the array size in state-of-the-art memristor-based neuromorphic computing is small, limiting the practical applications of memristive computing systems. To achieve large-scale arrays, a stable and uniform resistive switching device is a basic requirement.^12^ Moreover, sneak path issue is a severe challenge caused by the leakage current from undesignated cells in an array, which causes a limitation of array size and read/write errors. To overcome the sneak path issue, selection devices (selector) such as diode,^13^ threshold switching device,^14–20^ tunnelling device^21–24^ are proposed in series with the memristor device for configuring a one selector one resistor (1S1R) architecture. Ta/TaO_*x*_ is a widely accepted material system for resistive switching devices. The memristors based on Ta/TaO_*x*_ system shows excellent electrical characteristics, such as high endurance (more than 10^11^), fast speed (10 ns), low switching energy (sub 110 nJ), analogue switching.^25–28^ And the non-linear selector devices based on TaO_*x*_ have been reported before.^29–31^ However, a single device based on Ta/TaO_*x*_ system with coexistence of stable resistive switching and uniform non-linear selection has not yet been reported and a clear physical explanation of this mechanism is still lacking.

In this work, we investigate the coexistence of non-linear selection and resistive switching behaviors in Ta/TaO_*x*_/Ru device. The device shows uniform selection feature with a 10^3^ nonlinearity, 25 kA cm^−2^ ON-current density, fast speed, very low variation, and good endurance. The successful depression of the leaky current and increment of nonlinearity was confirmed by measuring the integrated 1S1R cell. Moreover, after applying a large bias on the Ta electrode, the device shows stable resistive switching feature. We presented a simple model to explain this phenomenon. The non-linear selection behavior may be attributed to a trapezoidal band structure modulated by the oxygen concentration gradient in the TaO_*x*_ layer formed in oxidation process of the Ta bottom electrode. And conductive filaments of probably Ta metals and oxygen vacancies formed under a large bias in the TaO_*x*_ layer cause the bipolar resistive switching behavior. The coexistence of non-linear selection behavior and resistive switching behavior in a single device deepens our understandings of the Ta/TaO_*x*_ material system, provides the flexibility to control the device for its intended use, and paves the way for further large-scale integration.

## Experiments

**Fig. 1:**
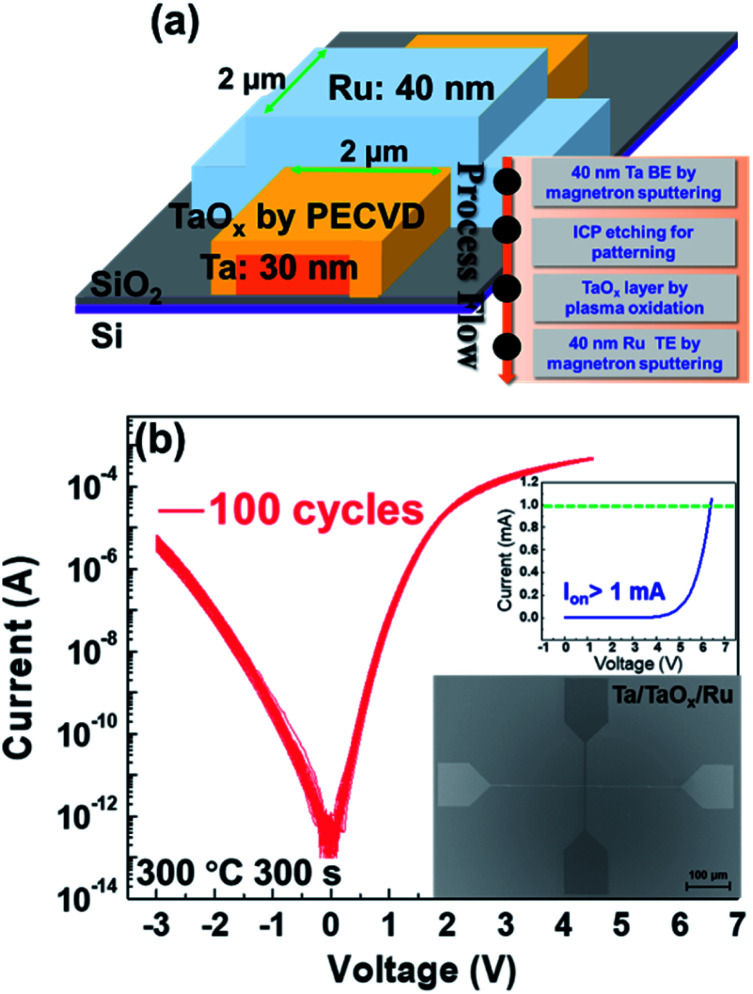
(a) Schematic diagram of the devices and the process flow of the devices. (b) *I*–*V* characteristics of the Ta/TaO_*x*_/Ru device as selector plotted in semi-log scale and linear scale (inset). Inset: SEM image of the Ta/TaO_*x*_/Ru device. (Oxidation parameters: 300 °C, 300 s.)

The Ta/TaO_*x*_/Ru device with crossbar structure was fabricated. A schematic of the device structure and SEM image of the device are shown in the Fig. 1, and the inset of Fig. 1(a) shows the process flow. Initially, a 30 nm thick Ta layer as bottom electrode was deposited on the SiO_2_/Si substrate through magnetron sputtering at room temperature. Secondly, the bottom electrode was patterned by Inductively Coupled Plasma (ICP) etching using C_3_F_8_ and Ar at 100 W with 100 s after the photolithography process. Subsequently, a TaO_*x*_ layer was formed by rapid thermal annealing (RTA) (300 s, O_2_ flow: 100 sccm) in plasma O_2_ at 300 °C. Direct oxygen plasma with a power of 100 W was applied on the Ta film. Finally, 40 nm Ru as the top electrode was deposited by magnetron sputtering followed by subsequent photolithography and lift-off processes. The direct current (DC) electrical characteristics of the device were measured by Agilent B1500A semiconductor parameter analyser. For pulse measurement, the pulse signals are applied by the WGFMU in B1500A analyser. And all the electrical characteristics were measured at room temperature. The size of the electrically tested devices is 2 μm × 2 μm. During the electrical measurement, the voltage was applied to the top Ru electrode, while the Ta bottom electrode was tied to ground.

## Result and discussion

**Fig. 2:**
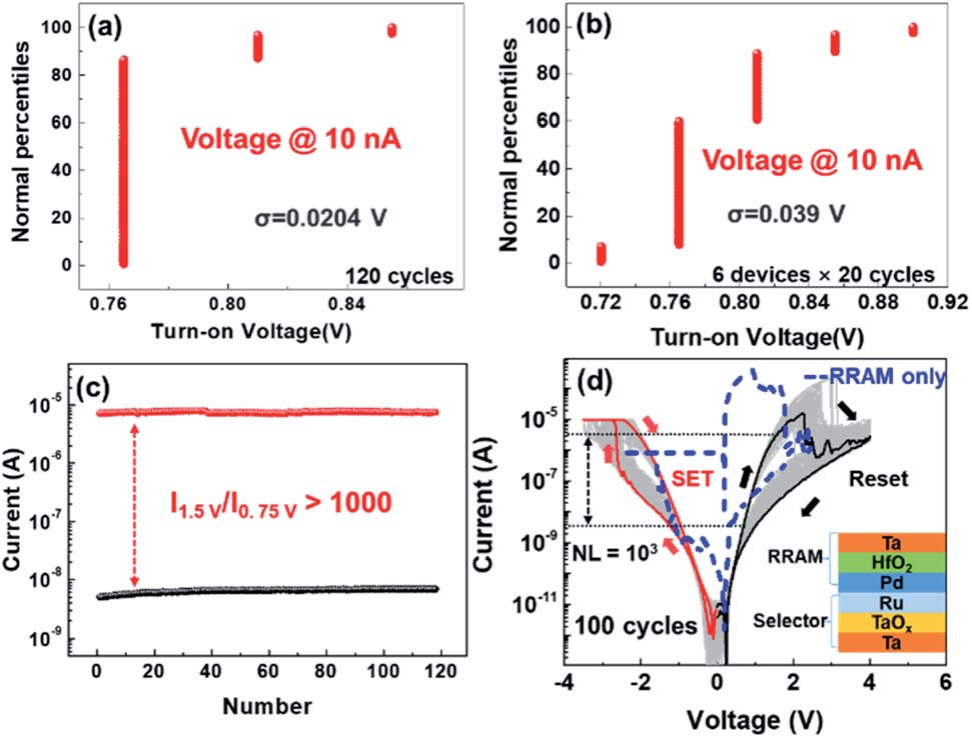
(a) Cumulative probability of the turn-on voltage for 120 cycles. (b) Cumulative probability of the turn-on voltage for 6 different devices with 20 cycles. (c) The current at *V*_read_ (1.5 V) and 1/2*V*_read_ (0.75 V) for 120 cycles. (d) *I*–*V* curves of the 1S1R cell for 100 cycles. (Inset: the structure of 1S1R cell.)

Typical *I*–*V* curves of the Ta/TaO_*x*_/Ru device tested by DC with 100 cycles and plotted in semi-log scale and linear scale are shown in Fig. 1(b). The *I*–*V* curves are overlapped with each other and only show very slight variations, indicating excellent cycle-to-cycle uniformity. The positive current is larger than 1 mA with a larger voltage as shown in the inset of Fig. 1(b), which means a high on-current density of 25 kA cm^−2^. The non-linearity (NL) is defined as NL = *I*_*V*read_/*I*_1/2*V*read_.^32^ The NL of the device is up to 10^3^ between 0.75 V and 1.5 V. The statistics are performed to further explored the uniformity of our device, as shown in Fig. 2(a). The states are defined as ON state when the current is upon 10 nA and defined as OFF state when the current is below 10 nA. Fig. 2(a) shows the probability of the turn-on voltage for one device with 120 cycles. The standard deviation is only 0.0204 V. To study the device-to-device uniformity of our selector, the probabilities of the turn on voltage for 6 different devices with 20 cycles are shown in Fig. 2(b), in which the standard deviation is only 0.039 V. The currents at *V*_read_, 1/2*V*_read_, also show a negligible deviation after 120 cycles as shown in Fig. 2(c), which implies that our devices have stable electrical characteristics and excellent uniformity. The NL of the device is maintaining at 10^3^ between 0.75 V and 1.5 V for 120 cycles, which suggests good ability of our devices to suppress the leaky current at low bias and can help to overcome sneak path issues in a large array. Consequently, the devices have very good device-to-device and cycle-to-cycle uniformity and is a great candidate to be used as a selector in crossbar arrays to overcome sneak path issue. The mechanism of non-linear selection feature of our device will be discussed in detail later in the mechanism part. Furthermore, to investigate the effectiveness of the selector in 1S1R structure, a Ta/HfO_2_/Pd (30 nm/5 nm/30 nm) resistive switching device was integrated with the Ta/TaO_*x*_/Ru selection device to configure a 1S1R cell, as shown in the inset of Fig. 2(d). It is worth noting that the Ta/HfO_2_/Pd device only needs 1 μA compliance current during SET process which is low enough to avoid breakdown of the Ta/TaO_*x*_/Ru selector. Typical *I*–*V* curves of a single Ta/HfO_2_/Pd resistive switching device and 100 switching cycles of the integrated 1S1R cell are shown in the Fig. 2(d). The current of LRS was limited by the selector device. As shown in Fig. 2(d), nearly 10^3^ NL (*V*_read_ = 1.5 V, 1/2*V*_read_ = 0.75 V) can be achieved in the integrated 1S1R cell, which significantly reduced half-selected cells' leakage current.^27,33^

**Fig. 3:**
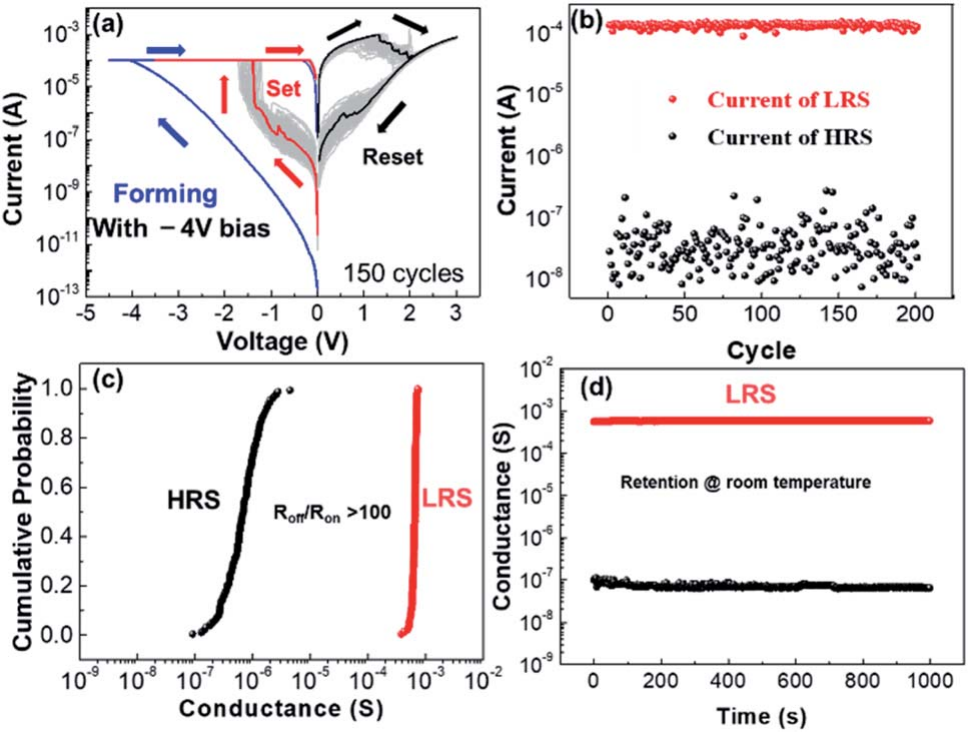
(a) Forming process of the device with a larger negative bias and the *I*–*V* curves of resistive switching after a lager negative forming for 150 cycles. (b) Resistive switching endurance test of 200 cycles. (c) Cumulative probability of LRS and HRS of 200 cycles. (c) Retention tests of the device at HRS and LRS. (d) Pulse operation of the device, the device shows fast switching speed. In SET process the switching speed is nearly 50 ns and in RESET process the switching speed is blow 150 ns.

**Fig. 4:**
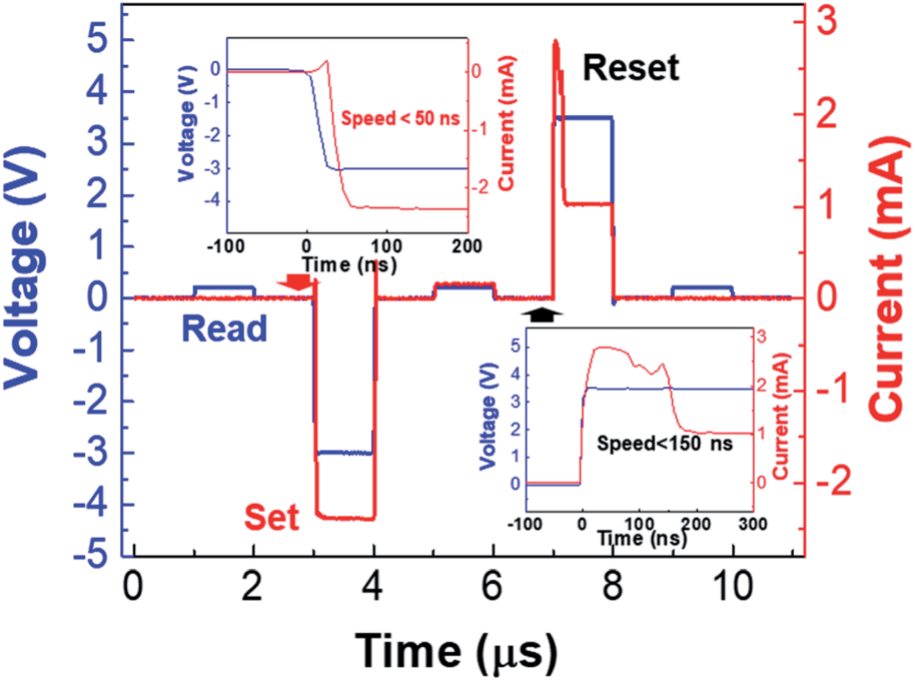
Pulse operation of the device, the device shows fast switching speed. In SET process the switching speed is nearly 50 ns and in RESET process the switching speed is blow 150 ns.

Besides the non-linear selection, our device also shows uniform resistive switching characteristics. As shown in Fig. 3(a), when increasing the negative bias on the Ru electrode beyond −4 V, a forming process and subsequently stable resistive switching behavior can be observed. The *I*–*V* curves of typical bipolar switching for 150 cycles with 100 μA compliance current at SET process are shown in Fig. 3(a). The SET voltages are at around −1.5 V and the RESET voltages are around 2 V. The stable resistive switching behavior of the device is demonstrated by a 200 cycle endurance test, as shown in Fig. 3(b). And the cumulative probabilities of the conductance at LRS and HRS for one device in 200 switching cycles are shown in the Fig. 3(c) with 0.2 V reading voltage. The *R*_on_/*R*_off_ ratio are beyond 100 with good uniformity. Fig. 3(d) shows that there is no obvious degradation of the states after 1000 s retention time at room temperature. Besides the DC sweeping, a series of pulses are applied to the device to prove that the device can be operated at very high speed, as shown in Fig. 4. The device is at HRS at first. Then voltage pulse sequence consists of 0.2 V read pulse, −3 V SET pulse, 0.2 V read pulse and 4 V RESET pulse are applied to the Ru electrode. The pulse width and interval of these pulse are all 1000 ns. When a programming pulse (SET or RESET pulse) is applied on the device, the resistance state of the device is switched. And the read pulse is used to monitor the resistance changes. The speeds of both SET and RESET processes are nearly 50 ns and 150 ns, respectively as shown in the inset of Fig. 4. Based on these results, the device can be used not only as a selector, but also as a memory with its uniform non-volatile resistive switching behavior and high switching speed.

**Fig. 5:**
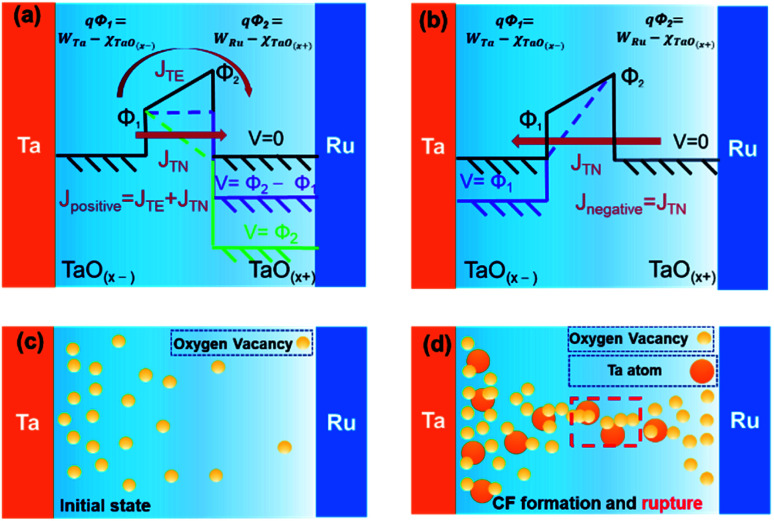
Schematic illustration of the mechanism of selection behavior and resistive switching behavior of our devices. (a) and (b) Band diagram of the device under positive and negative bias conditions. (c) The initial distribution of the oxygen vacancy. (d) Conduction filament formation after a large negative forming bias. The filament may rupture (RESET) and re-form (SET) in the red dashed box area.

Fig. 5 shows the proposed mechanism of non-linear selection and resistive switching behaviors of our devices. The asymmetric non-linear behavior can be attributed to the thermionic emission and tunnelling through a trapezoidal barrier. The barrier height on each side can be obtained by the work function of electrodes and TaO_*x*_ electron affinity (*χ* = 3.3 eV)^34^. As shown in Fig. 5(a), the formation of trapezoidal barrier (*qΦ*_1_ on Ta side, and *qΦ*_2_ on Ru side) originate in the work function difference between different electrodes and concentration gradient of oxygen caused by the oxidation process. As the plasma O_2_ is applied on the surface, the surface of the Ta bottom electrode is oxidized, however, the material under the surface may not be fully oxidized. As a result, a concentration gradient of oxygen may be achieved which leads to the formation of a trapezoidal barrier. Besides, due to the work function of Ru (*W*_Ru_ = 4.71 eV)^35^ is larger than Ta (*W*_Ta_ = 4.23 eV),^35^ the difference between barrier on Ru side (*Φ*_2_) and barrier on Ta side (*Φ*_1_) is further increased. When a positive bias is applied on the top electrode, both thermionic emission and tunnelling contribute to the electronic conduction. As the voltage increases, both tunnelling flux *J*_TN_ and the thermionic emission flux *J*_TE_ increase, owing to the decrease of highest part (*qΦ*_2_) of the energy band. Therefore, a high nonlinearity selection behavior can be achieved at positive bias. When a negative bias is applied, the tunnelling should be the dominant factor because the highest part of the energy band will not change, which cause the asymmetric *I*–*V* curves of the selection behavior, as shown in Fig. 5(b).

As shown in Fig. 5(c), most oxygen vacancies are disturbed on the Ta electrode side in the initial state. When a large bias is applied on the Ta electrode, the resistive switching behavior can be observed. Based on literature,^36^ both the Ta atoms and oxygen vacancies formed by the oxidation process participated in the resistive switching behavior. When a large forming bias is applied on the Ta bottom electrode, the oxygen vacancies, and Ta cations drift to the Ru top electrode. It has been demonstrated that the mobility of Ta cations within the oxide layers is comparable to that of the oxygen ions.^37^ As a result, oxygen vacancy and Ta conductive filaments are formed and switch the device to LRS, as shown in Fig. 5(d). During the RESET process, a negative bias is applied on the Ta electrode, the oxygen vacancies and Ta cations are drift back towards the Ta electrode, rupturing the conductive filaments, as a result, the device is switched from LRS to HRS. During the SET process, a positive bias is applied on the Ta electrode, the filaments are re-formed, and the device is switched from HRS to LRS.

## Conclusions

In conclusion, we investigate the coexistence of non-linear selection and resistive switching characteristics in Ta/TaO_*x*_/Ru device. The device can act as a uniform selector with 10^3^ nonlinearity, 25 kA cm^−2^ current density, high uniformity and its selection function is confirmed by a 1S1R integrated cell. Meanwhile, a stable resistive switching behavior is also achieved. Besides, we provide a sample model to explain our device behavior that the nonlinear selection behavior is attributed to the trapezoidal barrier, and the resistive switching behavior is mainly originated from the formation and rupture of the conductive filaments. These results may deepen our understanding for the Ta/TaO_*x*_ material systems and provide guidance for device optimalization and design.

## Conflicts of interest

There are no conflicts to declare.

## Supplementary Material
